# *rac*-Dichlorido[3-eth­oxy-3-(1-ethyl-1*H*-benzimidazol-2-yl)-2,3-dihydro-1*H*-pyrrolo­[1,2-*a*]benzimidazole]­copper(II)

**DOI:** 10.1107/S1600536812051641

**Published:** 2013-01-09

**Authors:** Robert T. Stibrany, Joseph A. Potenza

**Affiliations:** a Department of Chemistry and Chemical Biology, Rutgers, The State University of New Jersey, 610 Taylor Road, Piscataway, New Jersey 08854, USA

## Abstract

The title complex, [CuCl_2_(C_21_H_22_N_4_O)], contains a bis­(benzimidazole) unit with a chiral bridgehead C atom that forms part of a tetra­hydro­pyrrole ring fused to one of the benzimidazoles. The chelate angle is 90.45 (9)° and the dihedral angle between the essentially planar benzimidazole fragments is 26.68 (9)°. The Cu^II^ coordination geometry lies approximately midway between tetra­hedral and square planar. Overall, each chiral mol­ecule contains six fused rings, and a racemic mixture is formed with symmetry-related enanti­omers. In the crystal, C—H⋯π and C—H⋯Cl inter­actions link mol­ecules into a supra­molecular chain along the *a*-axis direction.

## Related literature

For ^19^F NMR studies of related compounds, see: Stibrany (2003 ▶). For polymerization studies, see: Stibrany *et al.* (2003 ▶). For their use as agents to study electron transfer, see: Knapp *et al.* (1990 ▶). For related structures, see: Baugh *et al.* (2006 ▶); Stibrany (2009 ▶); Stibrany *et al.* (2002 ▶, 2004 ▶); Stibrany & Potenza (2006 ▶, 2008 ▶). For calculation of the four-coordination geometry, see: Yang *et al.* (2007 ▶).
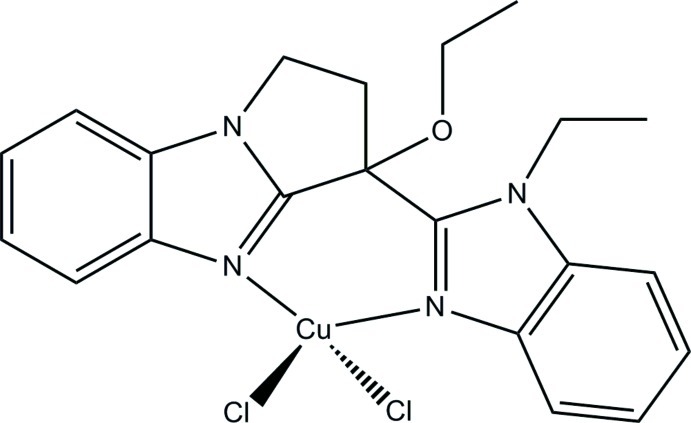


## Experimental

### 

#### Crystal data

[CuCl_2_(C_21_H_22_N_4_O)]
*M* *_r_* = 480.87
Triclinic, 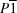

*a* = 8.9409 (17) Å
*b* = 9.5209 (18) Å
*c* = 14.323 (3) Å
α = 106.973 (4)°
β = 92.373 (4)°
γ = 113.778 (4)°
*V* = 1049.3 (3) Å^3^
*Z* = 2
Mo *K*α radiation
μ = 1.32 mm^−1^
*T* = 294 K
0.43 × 0.23 × 0.06 mm

#### Data collection

Bruker SMART CCD area-detector diffractometer
Absorption correction: multi-scan (*SADABS*; Bruker, 2000; ▶ Blessing, 1995 ▶) *T*_min_ = 0.771, *T*_max_ = 1.00
10062 measured reflections
4126 independent reflections
3380 reflections with *I* > 2σ(*I*)
*R*_int_ = 0.027

#### Refinement

*R*[*F* ^2^ > 2σ(*F* ^2^)] = 0.044
*wR*(*F* ^2^) = 0.124
*S* = 1.00
4126 reflections
264 parameters
1 restraint
H-atom parameters constrained
Δρ_max_ = 0.90 e Å^−3^
Δρ_min_ = −0.29 e Å^−3^

### 

Data collection: *SMART* (Bruker, 2000 ▶); cell refinement: *SAINT* (Bruker, 2000 ▶); data reduction: *SAINT*; program(s) used to solve structure: *SHELXS97* (Sheldrick, 2008 ▶); program(s) used to refine structure: *SHELXL97* (Sheldrick, 2008 ▶); molecular graphics: *ORTEPIII* (Burnett & Johnson, 1996 ▶), *ORTEP-3 for Windows* (Farrugia, 2012 ▶); software used to prepare material for publication: *SHELXTL* (Sheldrick, 2008 ▶).

## Supplementary Material

Crystal structure: contains datablock(s) I, global. DOI: 10.1107/S1600536812051641/tk5184sup1.cif

Structure factors: contains datablock(s) I. DOI: 10.1107/S1600536812051641/tk5184Isup2.hkl

Additional supplementary materials:  crystallographic information; 3D view; checkCIF report

## Figures and Tables

**Table:** 

Cu—N23	1.993 (2)
Cu—N13	2.005 (2)
Cu—Cl1	2.2169 (9)
Cu—Cl2	2.2198 (9)

**Table:** 

N23—Cu—N13	90.45 (9)
N23—Cu—Cl1	141.12 (8)
N13—Cu—Cl1	94.14 (7)
N23—Cu—Cl2	100.17 (7)
N13—Cu—Cl2	143.67 (8)
Cl1—Cu—Cl2	98.64 (4)

**Table 2:** Hydrogen-bond geometry (Å, °) *Cg*1 is the centroid of the C11/C13–C17 phenyl ring.

*D*—H⋯*A*	*D*—H	H⋯*A*	*D*⋯*A*	*D*—H⋯*A*
C4—H4*B*⋯*Cg*1^i^	0.96	2.99	3.910 (5)	160
C17—H17⋯Cl1^ii^	0.93	2.78	3.694 (4)	169

Symmetry codes: (i) 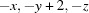; (ii) 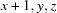.

**Fig. 1.**
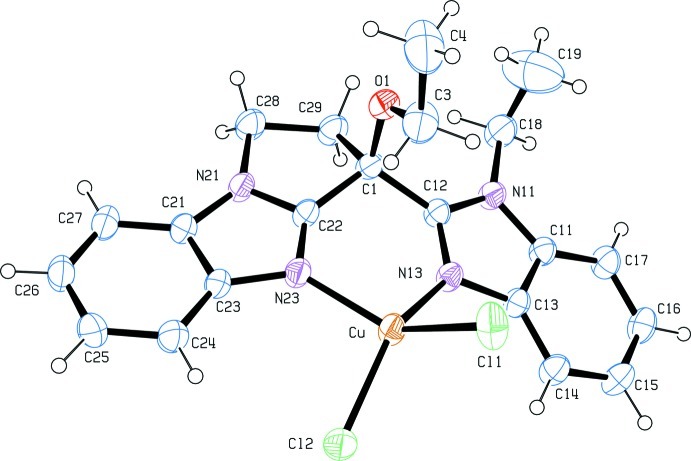
The molecular structure of the title compound (I) showing the atom-numbering scheme. Displacement ellipsoids are shown at the 40% probability level. H atoms are shown as spheres of arbitrary radius.

**Fig. 2.**
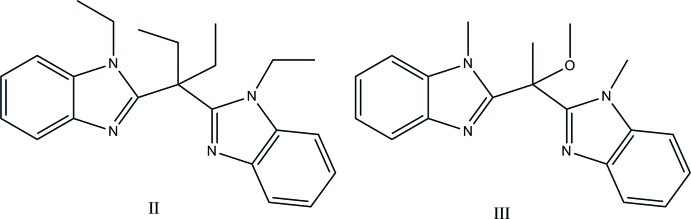
Drawings of previously reported quaternary substituted bis(benzimidazoles).

## supplementary crystallographic information

### Comment

The title complex (I), Fig. 1, was prepared as part of our long-term interest in the chemistry of bis(imidazoles), bis(benzimidazoles), and their complexes with metal ions. These species have demonstrated their usefulness as proton sponges (Stibrany *et al.*, 2002), geometrically constraining ligands (Stibrany *et al.*, 2004), agents to study electron transfer (Knapp *et al.*, 1990), polymerization catalysts (Stibrany *et al.*, 2003), ^19^F NMR polymerization catalyst probes (Stibrany, 2003), and in the formation of metal-organic copolymers (Stibrany & Potenza, 2008). In this study we extend the ring system with the addition of a fused tetrahydropyyrole.

Only two bis(benzimidazole) ligands containing quaternary bridgehead carbon atoms have been structurally characterized (Fig. 2) II (Stibrany, 2009) and III (Stibrany *et al.*, 2003; Stibrany & Potenza, 2006). Several structures containing bis(benzimidazole) ligands with a single bridgehead carbon atom of the form Cu^II^N_2_*X*_2_, where *X* is a halogen, have previously been reported (Baugh *et al.*, 2006; Stibrany, 2009; Stibrany *et al.*, 2003; Stibrany & Potenza, 2006; Stibrany & Potenza, 2008). Of those structures, several contain tertiary bridgehead carbon atoms (3') and the remaining contain quaternary bridgehead carbon atoms (4'). The "bite" angle of the bis(benzimidazole) ligands, which is defined as the N—Cu—N angle and is constrained by the ligand structure. The previously reported average for structures containing (4') carbon bridgehead atoms was reported as 90.4 (8)° (Stibrany, 2009). This compares favorably with the title structure which is 90.45 (9)° for the N23—Cu—N13 bond angle. The essentially planar benzimidazole fragments are twisted by 26.68 (9)°. A τ_4_ value of 0.53 indicates the coordination geometry is approximately midway between a perfect tetrahedral coordination geometry (τ_4_ = 1) and a perfect square-planar geometry (τ_4_ = 0) (Yang *et al.*, 2007).

### Experimental

In a 50 ml Erlenmeyer flask containing acetonitrile (10 ml), CuCl_2_^.^2H_2_O (20 mg) was dissolved. Then *rac*-[3-ethoxy-3-(1-ethylbenzimidazol-2-yl)-4,5- dihydro-pyrrolo[1,2-*a*]benzimidazole] (41 mg) was added to the flask to give a green solution. The flask was sealed in a jar containing diethyl ether designed to allow slow vapor diffusion of diethyl ether. After 3 days, yellow-green plates of the title complex formed.

### Refinement

Hydrogen atoms were positioned geometrically using a riding model, with C—H = 0.97 secondary alkyl, 0.96 primary alkyl, and 0.93 Å, and with *U*_iso_(H) = 1.2–1.5*U*_eq_ (C).

### Crystal data

**Table:**

[CuCl_2_(C_21_H_22_N_4_O)]	*Z* = 2
*M**_r_* = 480.87	*F*(000) = 494
Triclinic, *P*1	*D*_x_ = 1.522 Mg m^−^^3^
Hall symbol: -P 1	Mo *K*α radiation, λ = 0.71073 Å
*a* = 8.9409 (17) Å	Cell parameters from 897 reflections
*b* = 9.5209 (18) Å	θ = 5.0–50.8°
*c* = 14.323 (3) Å	µ = 1.32 mm^−^^1^
α = 106.973 (4)°	*T* = 294 K
β = 92.373 (4)°	Cleaved plate, yellow-green
γ = 113.778 (4)°	0.43 × 0.23 × 0.06 mm
*V* = 1049.3 (3) Å^3^	

### Data collection

**Table:**

Bruker SMART CCD area-detector diffractometer	4126 independent reflections
Radiation source: fine-focus sealed tube	3380 reflections with *I* > 2σ(*I*)
Graphite monochromator	*R*_int_ = 0.027
φ and ω scans	θ_max_ = 26.1°, θ_min_ = 2.4°
Absorption correction: multi-scan (*SADABS*; Bruker, 2000; Blessing, 1995)	*h* = −11→11
*T*_min_ = 0.771, *T*_max_ = 1.00	*k* = −11→11
10062 measured reflections	*l* = −17→17

### Refinement

**Table:**

Refinement on *F*^2^	Primary atom site location: structure-invariant direct methods
Least-squares matrix: full	Secondary atom site location: difference Fourier map
*R*[*F*^2^ > 2σ(*F*^2^)] = 0.044	Hydrogen site location: inferred from neighbouring sites
*wR*(*F*^2^) = 0.124	H-atom parameters constrained
*S* = 1.00	*w* = 1/[σ^2^(*F*_o_^2^) + (0.0832*P*)^2^ + 0.250*P*] where *P* = (*F*_o_^2^ + 2*F*_c_^2^)/3
4126 reflections	(Δ/σ)_max_ = 0.001
264 parameters	Δρ_max_ = 0.90 e Å^−^^3^
1 restraint	Δρ_min_ = −0.29 e Å^−^^3^

### Special details

**Table:**

Geometry. All e.s.d.'s (except the e.s.d. in the dihedral angle between two l.s. planes) are estimated using the full covariance matrix. The cell e.s.d.'s are taken into account individually in the estimation of e.s.d.'s in distances, angles and torsion angles; correlations between e.s.d.'s in cell parameters are only used when they are defined by crystal symmetry. An approximate (isotropic) treatment of cell e.s.d.'s is used for estimating e.s.d.'s involving l.s. planes.
Refinement. Refinement of *F*^2^ against ALL reflections. The weighted *R*-factor *wR* and goodness of fit *S* are based on *F*^2^, conventional *R*-factors *R* are based on *F*, with *F* set to zero for negative *F*^2^. The threshold expression of *F*^2^ > σ(*F*^2^) is used only for calculating *R*-factors(gt) *etc*. and is not relevant to the choice of reflections for refinement. *R*-factors based on *F*^2^ are statistically about twice as large as those based on *F*, and *R*- factors based on ALL data will be even larger.

### Fractional atomic coordinates and isotropic or equivalent isotropic displacement parameters (Å^2^)

**Table:**

	*x*	*y*	*z*	*U*_iso_*/*U*_eq_	
Cu	−0.05985 (4)	1.12548 (4)	0.28616 (3)	0.04148 (15)	
Cl1	−0.11321 (11)	1.17616 (13)	0.15011 (7)	0.0642 (3)	
Cl2	−0.17411 (11)	1.26316 (11)	0.38646 (7)	0.0588 (2)	
O1	0.0067 (3)	0.7244 (3)	0.15123 (16)	0.0505 (5)	
N11	0.3553 (3)	1.0483 (3)	0.21160 (19)	0.0420 (6)	
N13	0.1575 (3)	1.1244 (3)	0.25783 (17)	0.0375 (5)	
N21	−0.0518 (3)	0.7479 (3)	0.36364 (18)	0.0410 (5)	
N23	−0.1091 (3)	0.9408 (3)	0.33722 (18)	0.0411 (5)	
C1	0.0961 (3)	0.8415 (3)	0.2462 (2)	0.0385 (6)	
C3	−0.0847 (5)	0.7703 (5)	0.0917 (3)	0.0678 (10)	
H3A	−0.0093	0.8600	0.0729	0.081*	
H3B	−0.1594	0.8052	0.1287	0.081*	
C4	−0.1818 (6)	0.6250 (7)	0.0011 (3)	0.1055 (19)	
H4A	−0.1073	0.5879	−0.0329	0.158*	
H4B	−0.2395	0.6545	−0.0421	0.158*	
H4C	−0.2607	0.5393	0.0203	0.158*	
C11	0.4096 (3)	1.2079 (3)	0.2130 (2)	0.0397 (6)	
C12	0.2047 (3)	1.0057 (3)	0.2399 (2)	0.0365 (6)	
C13	0.2862 (3)	1.2549 (3)	0.2426 (2)	0.0369 (6)	
C14	0.3060 (4)	1.4126 (4)	0.2565 (2)	0.0466 (7)	
H14	0.2250	1.4460	0.2777	0.056*	
C15	0.4509 (4)	1.5166 (4)	0.2374 (3)	0.0513 (8)	
H15	0.4679	1.6228	0.2455	0.062*	
C16	0.5728 (4)	1.4675 (4)	0.2062 (2)	0.0516 (8)	
H16	0.6685	1.5415	0.1933	0.062*	
C17	0.5561 (4)	1.3137 (4)	0.1939 (2)	0.0483 (7)	
H17	0.6383	1.2816	0.1738	0.058*	
C18	0.4450 (4)	0.9499 (4)	0.1798 (3)	0.0625 (10)	
H18A	0.5629	1.0211	0.1924	0.075*	
H18B	0.4279	0.8787	0.2189	0.075*	
C19	0.3921 (9)	0.8517 (9)	0.0753 (4)	0.145 (3)	
H19A	0.2790	0.7719	0.0638	0.217*	
H19B	0.4620	0.7976	0.0563	0.217*	
H19C	0.4004	0.9206	0.0365	0.217*	
C21	−0.1714 (3)	0.7566 (3)	0.4191 (2)	0.0407 (6)	
C22	−0.0218 (3)	0.8560 (3)	0.3164 (2)	0.0375 (6)	
C23	−0.2082 (3)	0.8786 (3)	0.4025 (2)	0.0397 (6)	
C24	−0.3296 (4)	0.9138 (4)	0.4451 (3)	0.0535 (8)	
H24	−0.3543	0.9952	0.4356	0.064*	
C25	−0.4141 (4)	0.8241 (4)	0.5026 (3)	0.0575 (8)	
H25	−0.4979	0.8445	0.5314	0.069*	
C26	−0.3756 (4)	0.7040 (4)	0.5180 (2)	0.0526 (8)	
H26	−0.4345	0.6463	0.5572	0.063*	
C27	−0.2544 (4)	0.6675 (4)	0.4778 (2)	0.0497 (7)	
H27	−0.2288	0.5876	0.4890	0.060*	
C28	0.0448 (4)	0.6532 (4)	0.3416 (3)	0.0513 (8)	
H28A	−0.0222	0.5433	0.2962	0.062*	
H28B	0.0945	0.6486	0.4015	0.062*	
C29	0.1774 (4)	0.7556 (4)	0.2925 (3)	0.0492 (7)	
H29A	0.2095	0.6860	0.2417	0.059*	
H29B	0.2755	0.8354	0.3416	0.059*	

### Atomic displacement parameters (Å^2^)

**Table:**

	*U*^11^	*U*^22^	*U*^33^	*U*^12^	*U*^13^	*U*^23^
Cu	0.0349 (2)	0.0438 (2)	0.0595 (3)	0.02188 (17)	0.01855 (16)	0.02833 (18)
Cl1	0.0509 (5)	0.0988 (7)	0.0767 (6)	0.0443 (5)	0.0264 (4)	0.0576 (5)
Cl2	0.0677 (5)	0.0548 (5)	0.0766 (6)	0.0398 (4)	0.0346 (4)	0.0319 (4)
O1	0.0545 (13)	0.0378 (11)	0.0488 (12)	0.0117 (10)	0.0105 (10)	0.0121 (9)
N11	0.0327 (12)	0.0374 (13)	0.0589 (15)	0.0155 (10)	0.0175 (10)	0.0186 (11)
N13	0.0279 (11)	0.0363 (12)	0.0499 (13)	0.0122 (10)	0.0101 (9)	0.0187 (10)
N21	0.0402 (13)	0.0358 (12)	0.0511 (14)	0.0155 (10)	0.0124 (10)	0.0215 (11)
N23	0.0384 (13)	0.0376 (13)	0.0543 (14)	0.0172 (11)	0.0173 (11)	0.0227 (11)
C1	0.0362 (14)	0.0312 (14)	0.0483 (15)	0.0146 (12)	0.0098 (11)	0.0133 (12)
C3	0.063 (2)	0.066 (2)	0.054 (2)	0.0090 (19)	−0.0028 (17)	0.0210 (18)
C4	0.091 (3)	0.102 (4)	0.058 (2)	−0.014 (3)	−0.003 (2)	0.020 (2)
C11	0.0328 (14)	0.0373 (15)	0.0468 (15)	0.0123 (12)	0.0087 (11)	0.0152 (12)
C12	0.0284 (13)	0.0365 (14)	0.0455 (15)	0.0133 (11)	0.0091 (11)	0.0157 (12)
C13	0.0304 (13)	0.0368 (14)	0.0425 (15)	0.0116 (11)	0.0056 (11)	0.0163 (12)
C14	0.0405 (16)	0.0413 (16)	0.0608 (19)	0.0179 (13)	0.0113 (13)	0.0209 (14)
C15	0.0447 (17)	0.0374 (16)	0.069 (2)	0.0128 (14)	0.0041 (15)	0.0222 (15)
C16	0.0336 (15)	0.0453 (17)	0.0633 (19)	0.0011 (13)	0.0041 (13)	0.0245 (15)
C17	0.0320 (15)	0.0505 (18)	0.0619 (19)	0.0141 (13)	0.0135 (13)	0.0233 (15)
C18	0.051 (2)	0.054 (2)	0.097 (3)	0.0285 (17)	0.0366 (19)	0.034 (2)
C19	0.128 (6)	0.139 (6)	0.143 (6)	0.072 (5)	0.030 (5)	−0.005 (5)
C21	0.0364 (14)	0.0371 (15)	0.0453 (15)	0.0114 (12)	0.0081 (12)	0.0157 (12)
C22	0.0338 (14)	0.0314 (14)	0.0451 (15)	0.0101 (11)	0.0074 (11)	0.0157 (12)
C23	0.0361 (14)	0.0333 (14)	0.0462 (16)	0.0103 (12)	0.0118 (12)	0.0150 (12)
C24	0.0511 (18)	0.0455 (18)	0.070 (2)	0.0229 (15)	0.0246 (16)	0.0241 (16)
C25	0.0498 (19)	0.054 (2)	0.067 (2)	0.0194 (16)	0.0293 (16)	0.0201 (17)
C26	0.0499 (18)	0.0497 (18)	0.0485 (17)	0.0088 (15)	0.0150 (14)	0.0211 (15)
C27	0.0501 (18)	0.0448 (17)	0.0517 (18)	0.0125 (14)	0.0109 (14)	0.0242 (14)
C28	0.0536 (18)	0.0479 (18)	0.068 (2)	0.0294 (16)	0.0194 (15)	0.0290 (16)
C29	0.0477 (17)	0.0435 (17)	0.068 (2)	0.0259 (14)	0.0170 (15)	0.0251 (15)

### Geometric parameters (Å, º)

**Table:**

Cu—N23	1.993 (2)	C14—C15	1.373 (4)
Cu—N13	2.005 (2)	C14—H14	0.9300
Cu—Cl1	2.2169 (9)	C15—C16	1.391 (5)
Cu—Cl2	2.2198 (9)	C15—H15	0.9300
O1—C3	1.426 (4)	C16—C17	1.366 (5)
O1—C1	1.428 (3)	C16—H16	0.9300
N11—C12	1.357 (3)	C17—H17	0.9300
N11—C11	1.390 (4)	C18—C19	1.452 (6)
N11—C18	1.454 (4)	C18—H18A	0.9700
N13—C12	1.321 (4)	C18—H18B	0.9700
N13—C13	1.393 (4)	C19—H19A	0.9600
N21—C22	1.338 (3)	C19—H19B	0.9600
N21—C21	1.373 (4)	C19—H19C	0.9600
N21—C28	1.464 (4)	C21—C27	1.392 (4)
N23—C22	1.318 (4)	C21—C23	1.405 (4)
N23—C23	1.407 (3)	C23—C24	1.375 (4)
C1—C12	1.504 (4)	C24—C25	1.386 (5)
C1—C22	1.505 (4)	C24—H24	0.9300
C1—C29	1.546 (4)	C25—C26	1.391 (5)
C3—C4	1.498 (6)	C25—H25	0.9300
C3—H3A	0.9700	C26—C27	1.365 (5)
C3—H3B	0.9700	C26—H26	0.9300
C4—H4A	0.9600	C27—H27	0.9300
C4—H4B	0.9600	C28—C29	1.545 (4)
C4—H4C	0.9600	C28—H28A	0.9700
C11—C13	1.386 (4)	C28—H28B	0.9700
C11—C17	1.390 (4)	C29—H29A	0.9700
C13—C14	1.391 (4)	C29—H29B	0.9700
			
N23—Cu—N13	90.45 (9)	C17—C16—C15	121.9 (3)
N23—Cu—Cl1	141.12 (8)	C17—C16—H16	119.0
N13—Cu—Cl1	94.14 (7)	C15—C16—H16	119.0
N23—Cu—Cl2	100.17 (7)	C16—C17—C11	116.5 (3)
N13—Cu—Cl2	143.67 (8)	C16—C17—H17	121.8
Cl1—Cu—Cl2	98.64 (4)	C11—C17—H17	121.8
C3—O1—C1	116.9 (2)	C19—C18—N11	112.5 (4)
C12—N11—C11	106.7 (2)	C19—C18—H18A	109.1
C12—N11—C18	129.1 (3)	N11—C18—H18A	109.1
C11—N11—C18	124.2 (2)	C19—C18—H18B	109.1
C12—N13—C13	106.1 (2)	N11—C18—H18B	109.1
C12—N13—Cu	130.38 (19)	H18A—C18—H18B	107.8
C13—N13—Cu	123.17 (18)	C18—C19—H19A	109.5
C22—N21—C21	107.9 (2)	C18—C19—H19B	109.5
C22—N21—C28	114.1 (2)	H19A—C19—H19B	109.5
C21—N21—C28	138.0 (3)	C18—C19—H19C	109.5
C22—N23—C23	104.6 (2)	H19A—C19—H19C	109.5
C22—N23—Cu	118.62 (19)	H19B—C19—H19C	109.5
C23—N23—Cu	136.46 (19)	N21—C21—C27	132.3 (3)
O1—C1—C12	112.2 (2)	N21—C21—C23	105.3 (2)
O1—C1—C22	110.5 (2)	C27—C21—C23	122.3 (3)
C12—C1—C22	110.3 (2)	N23—C22—N21	113.5 (3)
O1—C1—C29	104.6 (2)	N23—C22—C1	135.6 (3)
C12—C1—C29	118.8 (2)	N21—C22—C1	110.7 (2)
C22—C1—C29	99.6 (2)	C24—C23—C21	120.1 (3)
O1—C3—C4	107.8 (4)	C24—C23—N23	131.1 (3)
O1—C3—H3A	110.1	C21—C23—N23	108.8 (2)
C4—C3—H3A	110.1	C23—C24—C25	117.9 (3)
O1—C3—H3B	110.1	C23—C24—H24	121.1
C4—C3—H3B	110.1	C25—C24—H24	121.1
H3A—C3—H3B	108.5	C24—C25—C26	121.0 (3)
C3—C4—H4A	109.5	C24—C25—H25	119.5
C3—C4—H4B	109.5	C26—C25—H25	119.5
H4A—C4—H4B	109.5	C27—C26—C25	122.5 (3)
C3—C4—H4C	109.5	C27—C26—H26	118.8
H4A—C4—H4C	109.5	C25—C26—H26	118.8
H4B—C4—H4C	109.5	C26—C27—C21	116.2 (3)
C13—C11—C17	122.0 (3)	C26—C27—H27	121.9
C13—C11—N11	106.4 (2)	C21—C27—H27	121.9
C17—C11—N11	131.6 (3)	N21—C28—C29	100.3 (2)
N13—C12—N11	112.2 (2)	N21—C28—H28A	111.7
N13—C12—C1	122.2 (2)	C29—C28—H28A	111.7
N11—C12—C1	125.5 (2)	N21—C28—H28B	111.7
C11—C13—C14	120.9 (3)	C29—C28—H28B	111.7
C11—C13—N13	108.6 (2)	H28A—C28—H28B	109.5
C14—C13—N13	130.4 (3)	C28—C29—C1	106.2 (2)
C15—C14—C13	116.8 (3)	C28—C29—H29A	110.5
C15—C14—H14	121.6	C1—C29—H29A	110.5
C13—C14—H14	121.6	C28—C29—H29B	110.5
C14—C15—C16	121.9 (3)	C1—C29—H29B	110.5
C14—C15—H15	119.1	H29A—C29—H29B	108.7
C16—C15—H15	119.1		
			
N23—Cu—N13—C12	25.1 (3)	C14—C15—C16—C17	−0.8 (5)
Cl1—Cu—N13—C12	−116.3 (2)	C15—C16—C17—C11	0.8 (5)
Cl2—Cu—N13—C12	133.1 (2)	C13—C11—C17—C16	0.3 (5)
N23—Cu—N13—C13	−162.5 (2)	N11—C11—C17—C16	−177.3 (3)
Cl1—Cu—N13—C13	56.1 (2)	C12—N11—C18—C19	83.6 (5)
Cl2—Cu—N13—C13	−54.5 (3)	C11—N11—C18—C19	−92.7 (5)
N13—Cu—N23—C22	−15.8 (2)	C22—N21—C21—C27	176.2 (3)
Cl1—Cu—N23—C22	81.4 (2)	C28—N21—C21—C27	−2.1 (6)
Cl2—Cu—N23—C22	−160.9 (2)	C22—N21—C21—C23	−0.8 (3)
N13—Cu—N23—C23	156.1 (3)	C28—N21—C21—C23	−179.1 (3)
Cl1—Cu—N23—C23	−106.8 (3)	C23—N23—C22—N21	−1.5 (3)
Cl2—Cu—N23—C23	11.0 (3)	Cu—N23—C22—N21	172.72 (19)
C3—O1—C1—C12	−51.1 (3)	C23—N23—C22—C1	173.0 (3)
C3—O1—C1—C22	72.5 (3)	Cu—N23—C22—C1	−12.8 (4)
C3—O1—C1—C29	178.8 (3)	C21—N21—C22—N23	1.5 (3)
C1—O1—C3—C4	−174.3 (3)	C28—N21—C22—N23	−179.8 (3)
C12—N11—C11—C13	−0.1 (3)	C21—N21—C22—C1	−174.4 (2)
C18—N11—C11—C13	176.9 (3)	C28—N21—C22—C1	4.3 (3)
C12—N11—C11—C17	177.8 (3)	O1—C1—C22—N23	−86.1 (4)
C18—N11—C11—C17	−5.2 (5)	C12—C1—C22—N23	38.5 (4)
C13—N13—C12—N11	−1.3 (3)	C29—C1—C22—N23	164.2 (3)
Cu—N13—C12—N11	172.07 (19)	O1—C1—C22—N21	88.5 (3)
C13—N13—C12—C1	−178.6 (3)	C12—C1—C22—N21	−146.9 (2)
Cu—N13—C12—C1	−5.2 (4)	C29—C1—C22—N21	−21.1 (3)
C11—N11—C12—N13	0.9 (3)	N21—C21—C23—C24	177.6 (3)
C18—N11—C12—N13	−175.9 (3)	C27—C21—C23—C24	0.2 (5)
C11—N11—C12—C1	178.1 (3)	N21—C21—C23—N23	−0.1 (3)
C18—N11—C12—C1	1.3 (5)	C27—C21—C23—N23	−177.5 (3)
O1—C1—C12—N13	97.8 (3)	C22—N23—C23—C24	−176.4 (3)
C22—C1—C12—N13	−25.9 (4)	Cu—N23—C23—C24	11.0 (5)
C29—C1—C12—N13	−139.9 (3)	C22—N23—C23—C21	0.9 (3)
O1—C1—C12—N11	−79.1 (3)	Cu—N23—C23—C21	−171.7 (2)
C22—C1—C12—N11	157.2 (3)	C21—C23—C24—C25	−0.9 (5)
C29—C1—C12—N11	43.2 (4)	N23—C23—C24—C25	176.1 (3)
C17—C11—C13—C14	−1.4 (5)	C23—C24—C25—C26	1.0 (5)
N11—C11—C13—C14	176.7 (3)	C24—C25—C26—C27	−0.2 (5)
C17—C11—C13—N13	−178.8 (3)	C25—C26—C27—C21	−0.6 (5)
N11—C11—C13—N13	−0.7 (3)	N21—C21—C27—C26	−176.0 (3)
C12—N13—C13—C11	1.2 (3)	C23—C21—C27—C26	0.6 (4)
Cu—N13—C13—C11	−172.76 (18)	C22—N21—C28—C29	14.7 (3)
C12—N13—C13—C14	−175.9 (3)	C21—N21—C28—C29	−167.1 (3)
Cu—N13—C13—C14	10.1 (4)	N21—C28—C29—C1	−27.3 (3)
C11—C13—C14—C15	1.4 (5)	O1—C1—C29—C28	−84.9 (3)
N13—C13—C14—C15	178.2 (3)	C12—C1—C29—C28	149.1 (3)
C13—C14—C15—C16	−0.4 (5)	C22—C1—C29—C28	29.4 (3)

### Hydrogen-bond geometry (Å, º)

Cg1 is the centroid of the C11/C13–C17 phenyl ring.

**Table:**

*D*—H···*A*	*D*—H	H···*A*	*D*···*A*	*D*—H···*A*
C4—H4*B*···*Cg*1^i^	0.96	2.99	3.910 (5)	160
C17—H17···Cl1^ii^	0.93	2.78	3.694 (4)	169

Symmetry codes: (i) −*x*, −*y*+2, −*z*; (ii) *x*+1, *y*, *z*.
